# Medical and Philosophical News

**Published:** 1782

**Authors:** 


### SECTION III.

### MEDICAL and PHILOSOPHICAL NEWS.

AT a public meeting of the Royal Medical Society at Paris, on the 27th of Auguſt, the premium offered by the Society in 1780, for the beſt diſſertation on Dropſy, was divided between Profeſſor Camper and M. Baraillon, two of the candidates. The eſſays preſented by theſe gentlemen are ſaid to contain a great variety of uſeful and ingenious obſervations; but as they both both leave ſomething to be wiſhed for relative to the treatment of dropſy, the Society have propoſed the ſubject a ſecond time, in the following form, for a prize of 600 livres:

"What are the ſpecies and different caſes of "Dropſy, in which the uſe of diluents or a dry "regimen ought to be preferred?"

The diſſertations on this ſubject are to be written in French or Latin, and ſent to M. Vicq D'Azyr, ſecretary to the Society, before the 1ſt of January 1784.

The Society likewiſe offer a premium of 200 livres to any perſon who ſhall prove, by ſatisfactory experiments, whether the Scurvy is a contagious diſeaſe, or not. Diſſertations on this ſubject will be received till May 1, 1783.

At the above meeting were read the eulogium of the late Dr. Fothergill, one of the ſixty foreign aſſociates of the Society, by M. Vicq D'Azyr; an eſſay on the combination of kermes mineral with cauſtic fixed alkali, and its utility as a remedy, by M. Hallé; obſervations by M. de Laſſone and M. Cornette, on the ſolubility of mercurial precipitates in water, and on the combination of mercury with volatile alkali; and laſtly, remarks on the intermittents of 1780 and 81, by M. Caille.

The Academy of Sciences at Lyons have offered a premium of 600 livres, for an experimental inquiry which ſhall aſcertain the properties of alum diſſolved in wine, the means of detecting it, and of correcting its ill effects. The diſſertations are to be ſent to the Secretary before the 1ſt of April 1783.

---

At a public meeting held lately at the College of Pharmacy at Paris, M. Lunel read an eſſay on the means of procuring an emetic which ſhall never vary in its effects. The proceſs for this purpoſe conſiſts in dephlogiſticating glaſs of antimony with acid of vitriol, before it is combined with cream of Tartar.

---

M. Bonnel de la Bragereſſe has communicated lately to the Royal Society at Montpellier an account of the effects of ſome new remedies, and, amongſt others, of an extract of the *anemone pulſatilla* Linn. in cutaneous diſeaſes, adminiſtered in doſes of a grain and a half twice a day.

---

M. Sail-

M. Saillant has lately preſented to the College of Phyſicians at Paris, a ſpecimen of a new drug from Madagaſcar, which is ſaid to be of conſiderable efficacy in diarrhœa. It is a thin bark, of a yellowiſh colour externally, reddiſh within, and to the taſte ſlightly bitter and aſtringent. It is known at Madagaſcar by the name of *Beläe*.

---

The death of the Archbiſhop of Paris lately of a dropſy, and the difference in opinion relative to his treatment that took place between M. Bouvart and M. Bacher, the two phyſicians who attended him, has given riſe to two very long letters from the latter in the *Journal de Medecine* for Jan. and Feb. 1782, in which the conduct of M. Bouvart (who, by the bye, is the phyſician in the greateſt vogue at Paris) ſeems to be arraigned with unjuſtifiable ſeverity. It ſeems that, in the caſe in queſtion, M. Bouvart recommended abſtinence from liquids, while M. Bacher, who has deſervedly gained great reputation in dropſies by his pills and the uſe of diluents, adviſed the patient to drink freely of diluting liquors. This latter method was accordingly tried for a few days; but as the complaint was not alleviated by this practice, it was given up in in favour of M. Bouvart's mode of treatment. The Archbiſhop died ſoon after; and M. Bacher now goes ſo far as to ſay, that if his preſcriptions had been ſteadily adhered to, the prelate's life would probably have been ſaved, tho' from his own account of the appearances on diſſection, (ſetting aſide the very advanced age of the patient, who was in his 79th year) the diſeaſe ſeems clearly to have been in its nature incurable. Eight pints of water were found in the cavity of the abdomen; and the liver was much enlarged, and in a ſchirrous ſtate, as were likewiſe the omentum and pancreas. The Archbiſhop had alſo a fiſtula in ano, and had formerly been cut for the ſtone.

---

It has long been lamented, that our two univerſities of Oxford and Cambridge, though ſo deſervedly celebrated for their claſſical learning, and their excellence in various branches of ſcience, have hitherto never acquired any reputation as ſchools of phyſic, ſo that medical ſtudents have been under the neceſſity of reſorting to Edinburgh, or to foreign univerſities, for that inſtruction which they in vain wiſhed to procure at home. It is with ſingular pleaſure, therefore, therefore, we communicate to our readers the following extract of a letter from a very respectable physician at Oxford, as it will serve to shew, that the present medical professors of that university, animated with a liberal zeal for reformation and improvement, are exerting their endeavours to render it useful as a school of physic. We have no doubt but their endeavours will be crowned with success, and that in medical reputation Oxford will very soon vie with Edinburgh, Leyden, and Vienna.

"The right hon. George Henry earl of "Litchfield, late chancellor of this university, "dying in 1772, bequeathed, after the demise "of his lady, the sum which should arise from "the sale of his house in London, and the "furniture thereof, in trust, to the then chancellor of Oxford, the bishop of Oxford, and "the president of St. John's College, for the "establishment of a Clinical Professorship in the "Radcliffe Infirmary. Her ladyship died in the "year 1779; when the sale of the said house "and furniture produced, clear of all expence, "4256 l. which sum was vested in the *Three per* "*cent. Consol.* and purchased 7079 l. stock, the "interest whereof amounts annually to 212 l. "which is the salary of the professor, who by "the

" the noble benefactor's will is to be elected by

" the members of Convocation. No person is

" eligible who has not taken a doctor's degree

" in physic at least five years before such elec-

" tion, and by the statutes, drawn up by the

" present trustees, he is required to give con-

" stant daily attendance, with the pupils, on cer-

" tain select patients in the Infirmary, and twice

" in each week to read a lecture on their cases,

" during the winter months, from the beginning

" of November to the latter end of March.

" Dr. Parsons was unanimously elected the first

" professor in 1781. This establishment was

" intended by the founder for the promotion of

" the study of physic in Oxford; and, that his

" great design may not be frustrated, the Con-

" vocation have abridged the time required for

" taking degrees in physic, one year after ad-

" mission to regency in arts (instead of three)

" being now sufficient for the degree of bachelor

" of physic, and three years from the degree of

" bachelor to that of doctor, instead of four, as

" formerly. The physicians resident in the

" place have given their assistance to the design.

" Dr. Parsons continues to read two courses in

" anatomy every year. Dr. Wall reads two

" courses in chemistry. A course of lectures on

" the

" the practice of phyfic will next year be read
" by Dr. Vivian, and on phyfiology by Dr.
" Smith. The Rev. Mr. Shaw teaches botany,
" and Dr. Auftin delivers mathematical lectures.
" The number of ftudents in phyfic is already
" much increafed, and they have formed them-
" felves into a MEDICAL SOCIETY, which has
" regular weekly meetings during the winter
" months, and of which Martin Wall, M. D.
" is prefident, and William Auftin, M. B. vice-
" prefident."

---

The celebrated Haller, in his *Opufcula Pathologica*, after giving the hiftory of a young lady of quality who died at the age of fix-and-twenty, and who was found to have two uteri, each of an oval fhape and furnifhed with its own peculiar vagina, obferves, that a woman fo formed might certainly be liable to one conception upon the back of another, and therefore that a perfect fuperfœtation could take place in fuch a perfon. Similar remarks are made by Dr. Purcell, in his ingenious account of a double uterus, publifhed in the 64th volume of the Philof. Tranfactions. and indeed we have no doubt, that the cafes of fuperfœtation recorded by different writers, if they did actually occur, were owing to a conformation of this ſort, which has been noticed by medical writers only in a ſmall number of inſtances, and in theſe not till after death. But a caſe of ſuperfœtation having occurred lately to Dr. Lobſtein, profeſſor of anatomy and ſurgery at Straſburgh, in a woman who is ſtill living in that city, and who was delivered of two children, one a month after the other, he has been able to convince himſelf, that this circumſtance is owing to her having two uteri, each of which has a diſtinct vagina.——Dr. Lobſtein means to favour the public with a particular account of this curious fact.

---

Dr. Schotte (author of an ingenious treatiſe lately publiſhed on the ſynochus atrabilioſa) has communicated to the Royal Society an account of a remarkable inſtance of ſarcocele, which occurred to him at Senegal. The patient was a negro about fifty years old, and the diſeaſe had been gradually advancing for the ſpace of five-and-twenty years. It was attended with no pain, but the enormous bulk of the ſcrotum was of itſelf a ſufficient inconvenience, as it obliged the patient to lie conſtantly in bed. At the time Dr. Schotte ſaw it the urine had made its way through a fiſtulous ulcer *in perinæo*, and the integuments of the ſcrotum were hard and thickened.

ened. The ſcrotum itſelf he had not an opportunity of meaſuring, but he is certain that he ſpeaks within compaſs when he ſays, that it was in length two feet and a half, and in diameter, from thigh to thigh, eighteen inches.

This is the only inſtance of this diſeaſe that the Doctor himſelf has met with; but he has been informed, that in Galam, a country diſtant about 900 miles E. from Senegal, as well as in ſome other parts of Africa, it is endemial, and ſuppoſed to be hereditary.

---

From the report of the SMALL POX SOCIETY at CHESTER, dated Sept. 17, 1782, it appears, that of 213 children, inoculated by the Society, only two died; and it was doubted whether the death of either of theſe could be properly attributed to inoculation, as there was reaſon to ſuſpect, that one of the patients had previouſly received the natural infection; and the death of the other ſeemed to be occaſioned by a diſorder of the bowels. Of 203 private patients, who were inoculated during the laſt four years, not one died.

In one part of the town the inhabitants, diſregarding the inſpectors exhortations, purpoſely propagated the diſtemper, carrying the poiſon and even patients, from one houſe to another

 without without reserve. In consequence of this conduct it spread through fifteen families, and proved fatal to several. This irregularity in part proceeded from their ignorance, that some money might be obtained by observing the rules. The hope of procuring the reward has had some influence on their conduct; and at the time of dating the report, the infection was nearly extinguished, as the small pox was then only in four families.

Soon after the Society was established, the natural distemper began to spread so quickly in Handbridge, through the irregularity of the inhabitants of that part of the town, and the funds of the institution being at that time so low as to cause an apprehension, that the rewards of prevention might become too expensive, the regulations were suspended in that quarter for some months. During the suspension, 16 died of the natural small pox in this parish; and in the spring of 1780, the distemper was spread so widely by soldiers, as to occasion a general suspension of the regulations for some months; during this time the deaths by the natural distemper amounted to 58. Taking the whole period of four years, ending March 30, 1782, the small pox, at Chester, has been fatal to 139, or 35 annually.

nually. If we deduct the above-mentioned 16 and 58, who died during the interval when the regulations were not executed, the total deaths would be only 55, or 14 annually; whereas the annual average of deaths by this diftemper, for fix years previous to the eftablifhment of this Society, was 63. Hence its fatality has been actually reduced to near one-half; and, if we may deduct the number who died during the two periods, when the regulations were fufpended, to near one-fifth.

The infpectors were particulary inftructed to obferve, whether the natural fmall pox was received from inoculated patients. On the moft careful inquiry, one private and one or two public patients appeared to have communicated the natural diftemper; and in all thefe inftances, the perfons infected had unreferved intercourfe with the infectious, without any wifh to avoid them.

During the year 1781 only eight perfons died of this diftemper in Chefter, of whom two were infected in Manchefter, a third in Liverpool, and a child of the fourth in Coventry.

On mature confideration the Society have thought proper to abolifh the rewards to the inoculated patients, which at firft feemed neceffary to overcome inveterate prejudices.

---

Extract

Extract of a Letter from Dr. Vëling, physician at Aix la Chapelle, to Dr. Willan, physician in London.

" Opusculum tuum de *Lanuginosa Bysso* *, " quod dilectus amicus meus Weidmann, dum " hâc transiit, mihi præbuit, gaudio legi, et " quia non solum Aquisgrani sed etiam in pago " Burdscheid plurimi fontes aquam mineralem " calidam eructant, statim ad illos fontes nos " contulimus, et ecce! inveniebamus dictam " plantam a te descriptam, lapides eadem ratione " obtegentem. Sumo libertatem te illa de re " certiorem reddendi, et monstra mihi occasi- " onem ut tibi ad propriam inspectionem mittere " possim."

* Dr. Willan, in examining the mineral water at Croft (see page 327) discovered, by means of a microscope, that the white, hairy, mucous matter, which adheres to the sticks, grass, &c. in the course of that and other sulphur waters, is evidently a vegetable substance, answering to the character of Byssus, though a species not described in the system of Linnæus. Dr. Willan has named it *Byssus Lanuginosa*. It is a remarkable circumstance, that this Byssus is found below the spring no further than the water retains the sensible sulphureous qualities, as if the hepatic gas was necessary to its production and nourishment.

---

Mr.

Mr. John Hunter has lately communicated to the Royal Society an account of the organ of hearing in fishes.

---

At the anniverfary meeting of the Royal Society, on the 30th of November, the Copleian medal was conferred on Richard Kerivan, Efq. F. R. S. for his ingenious experiments and obfervations on the fpecific gravities and attractive powers of various faline fubftances. (See the 2d vol. of this Journal, page 191.)

---

Extract of a Letter from Dr. Hahn, Profeffor of Phyfic at Leyden, to Dr. Simmons (dated Oct. 3, 1782.)

" The Medical Society at the Hague mean
" foon to publifh the letters, relative to the late
" epidemic catarrh, which have been addreffed
" to them by the phyficians in different parts of
" the United Provinces. The phyficians at
" Haerlem intend to give their obfervations on
" the fame fubject in a feparate publication.
" Both thefe works will be in Dutch.

" The

"The Red Bark ſells at preſent at Amſter-

"dam for 50 ſols (not quite five ſhillings ſter-

"ling) a pound. Mr. Brand, one of our prin-

"cipal druggiſts, has lately ſupplied an Engliſh

"dealer with two thouſand pounds weight of it

"at that price."——The celebrated Profeſſor Camper, who has introduced the uſe of this Bark in Frieſland, and experienced its good effects in a variety of inſtances, obſerves, in a letter to Dr. Simmons, that ʒvj. of it are equal in efficacy to ℥j. of the common bark.—We had entertained hopes, that the Red bark was an eſtabliſhed article of commerce at Amſterdam, but we learn from Dr. Camper's letter, that the ſupply of it there was as accidental as in this country, and conſiſts only of a part of the cargo of a Spaniſh prize purchaſed at Liſbon. (*See page* 252).

---

*New Works about to be publiſhed.*——1. A treatiſe on exerciſe as a means of preſerving health, by M. Pinnel, phyſician at Paris. The author has been employed ſeveral years on this work. One of our friends at Paris, who has peruſed the MSS. and is a good judge of the ſubject, ſpeaks of it as a uſeful performance. It is ſoon to go to preſs.——2. A deſcription of the trees, ſhrubs, and plants of the Ruffian empire, which is to be publifhed by Profeffor Pallas, by order and under the aufpices of her Imperial Majefty. From the profpectus of this work, which has lately been fent to the Royal Society, it promifes to be one of the moft fplendid publications that has hitherto appeared on a botanical fubject. The whole is to be executed at the expence of the Emprefs. The plates are to be of the fame fize as thofe of Profeffor Jacquin's, and coloured after nature with the moft fcrupulous exactnefs. The defcriptions are to be in Ruffian and Latin. The number of plates will amount to about fix hundred. The work is to be publifhed in numbers. Each number is to contain fifty plates. No time is yet fixed for the appearance of the firft number; but when the publication is begun, it is propofed to bring out at leaft one number every year.——3. A fixth volume of the medical obfervations and inquiries is expected to be publifhed in a few months.——4. A fyftem of furgery is preparing for the prefs, in one large volume, 4to. by Mr. Fynney, of Leek in Staffordfhire. ——5. An account of a new operation in furgery in certain cafes, viz. the fawing off the extremities of the os femoris, tibia, and fibula, and uniting them by callus, fo as to form a ftiff joint,

 inftead instead of amputating the leg, is expected soon from Mr. Henry Park, one of the surgeons to the Infirmary at Liverpool.——6. M. Vicq D'Azyr is about to illustrate the anatomy of the brain by some very accurate engravings. About a dozen of the plates for this work, which is to be in folio, are already finished.——7. A new edition of Dr. Simmons's work on consumptions is preparing for the press, with considerable additions. Among other things it will contain farther remarks on the whiteness of the teeth as a mark of phthisis from tubercles.

