# Errata

**Published:** 1782

**Authors:** 


					p. 431, /. 6,' for Ken van rtcA,

				

